# Sleep quality and associated factors among pregnant women attending antenatal care at Jimma Medical Center, Jimma, Southwest Ethiopia, 2020: cross-sectional study

**DOI:** 10.1186/s12888-021-03483-w

**Published:** 2021-09-26

**Authors:** Tamrat Anbesaw, Habtamu Abebe, Chalachew Kassaw, Tilahun Bete, Alemayehu Molla

**Affiliations:** 1 Department of Psychiatry, College of Medicine and Health Science, Wollo University, P.O. Box 1145, Dessie, Ethiopia; 2 Department of Epidemiology and Biostatistics, Faculty of Medical science, Institute of Health Sciences, Jimma University, Jimma, Ethiopia; 3 Department of Psychiatry, College of Medicine and Health Science, Dilla University, P.O. Box 419, Dilla, Ethiopia; 4 Department of Psychiatry, College of Medicine and Health Science, Haramaya University, P.O. Box 235, Harar, Ethiopia

**Keywords:** Sleep quality, Pregnancy, Jimma, Ethiopia

## Abstract

**Background:**

Sleep is a natural physiological process vital for the physical and mental wellbeing of pregnant women and their fetuses. Even though poor sleep quality is a common problem among pregnant women, it is not studied in developing countries including Ethiopia. Therefore, this study was aimed to assess the poor sleep quality and associated factors among pregnant women attending antenatal care at Jimma medical center, Jimma, Southwest Ethiopia, 2020.

**Methods:**

A cross-sectional study design was conducted among 415 pregnant women at Jimma Medical Center (JMC). The study subjects were selected using a systematic random sampling technique. Pittsburgh Sleep Quality Index (PSQI) was used to assess sleep quality using face-to-face interviews. SPSS version 25 was used to analyze data. Bivariate and multivariable logistic regressions were done to identify factors related to sleep quality. In multivariable logistic regression variables with a *p*-value less than 0.05 was considered significant and, adjusted OR (AOR) with 95% CI was used to present the strength of the association.

**Results:**

The prevalence of poor sleep quality among pregnant women was found to be 30.8% (95% CI (26.5, 35.2). In multivariable analysis, age ≥ 30 years old (AOR = 1.94;95%CI:1.03,3.66), Multigravida (AOR = 1.90;95%CI:1.90,3.32),depression (AOR = 4.26;95%CI:2.54,7.14),stress (AOR = 1.85;95%CI:1.20,3.02) were variables significantly associated with poor sleep quality.

**Conclusion:**

This study found a high prevalence of poor sleep quality among pregnant women. Older age, gravidity, depression, and stress were associated with poor sleep quality. It is better to have routine sleep pattern screening and teach sleep hygiene practice for pregnant women.

## Introduction

Sleep is a physiological process and vital for the normal physical and mental well-being of an individual [1]. Sleep disorder is one of the most common type’s mental illnesses, directly related to the thoughts, feelings, and behaviors of humans. Pregnancy is the term given to a woman while her fetus develops inside her uterus [2]. It causes many physical, social, economic, psychological, and hormonal changes contributing to develop different forms of mental illness for a short and long period [3] . In the first trimester period of pregnancy, night urination, shortness of breath, heartburn, forced body position in bed, breast tenderness, and itching are the physiological changes affecting sleep [4]. Quality of sleep is essential for the physical and mental health of both the mother and fetus. A pregnant mother should get at least eight hours of night sleep [5]. The most common sleep problems observed among pregnant women were resting leg syndrome, sleep apnea, insomnia, nocturnal gastroesophageal reflux, and Sleep-related breathing problems [6]. Nearly, 78% of pregnant women’s had any forms of sleep disorder during their pregnancy time and complained of physical problems such as migraine headaches, gestational diabetes, obesity, cardiovascular conditions (hypertension and heart problems), and poor digestion [7]. A pregnant woman who slept less than six hours has a risk for premature birth, pre-eclampsia, prolonged labor, low progesterone level, abruption placenta, miscarriage, fetal death, and low birth weight [8, 9]. Almost 70% of mental health conditions including depression, suicidal ideations, and postpartum psychosis happened during pregnancy are due to subjective and objective sleep pattern problems [10]. The prevalence of poor sleep quality among pregnant women in the USA (53–71%) [11], China (87%) [12], and Iran (96.4%) [13].

The determinants of poor sleep quality among pregnant women were socio-economic status, age, divorce, body mass index, first pregnancy, history of fetal death, history of prolonged labor, zinc and fluorine deficiency, gestational period, and having a history of chronic medical illness (DM, hypertension), lack of awareness about sleep hygiene practice, unwanted pregnancy, lack of social support, and history of mental illness [2, 12, 14].

The most effective intervention for sleep problems related to pregnancy was basic sleep hygiene practice and progressive muscle relaxation techniques [15]. The use of medication for sleep problems related to pregnancy is the last option and better not used in the first trimester of pregnancy [16]. Despite the above prevalence and impact of sleep on the physical and mental well-being of both a mother and a fetus, there is limited study evidence in African countries including Ethiopia. Therefore, this study aimed to assess the prevalence of poor sleep quality and its correlates among pregnant women attending perinatal service at Jimma Medical Center, Southwest Ethiopia,2020.

## Methods and materials

### Study design and period

An institutional-based cross-sectional study was conducted in August 1–30/ 2020.

### Study area

The study was conducted at Jimma medical center (JMC) antenatal care units. JMC is located in Jimma town, Oromia regional state, which is found in the southern part of Ethiopia 325 km far away from Addis Ababa. The center gives service to the catchment population of about 15 million people. There was about 9850 pregnant mother who had a follow-up in a year at antenatal care and on average of a typical month 848 pregnant mother visit antenatal care for follow up.

### Source population

All pregnant women who had ANC follow up at Jimma Medical Center.

### Study population

Pregnant women attending the antenatal care follow up during the study period.

### Inclusion and exclusion criteria

#### Inclusion criteria

Women with a gestational age of 4 weeks and above, and aged 18 and above were included in this study.

#### Exclusion criteria

Women who were critically ill and difficult to communicate were excluded from the study.

### Sampling procedure and sampling techniques

#### Sample size estimation

The sample size was estimated by using a single population proportion formula. Sample size with z-value of 1.96 and marginal error of 5% sample was calculated as:

$$ n=\frac{{\left(\mathrm{Z}\;\upalpha /2\right)}^2\times \mathrm{P}\left(1\hbox{-} \mathrm{P}\right)}{{\mathrm{d}}^2} $$

Assumption: n = initial sample size need for this study *α* = confidence interval (95%) p = proportion of =50% (0.5) d = marginal error of 5%, (z α/_2_)^2 =^1.96.

$$ n=\frac{1.96^2\times \left(0.5\;\Big(1-0.5\right)=384}{(0.05)^2} $$

To calculate the sample size, 50% of the proportion was used since there is no related study done to identify the prevalence of poor sleep quality in Ethiopia. We added a 10% (39) nonresponse rate; finally, the total sample size was 423.

### Sampling procedure

Systematic random sampling was used to invite respondents within every two intervals while coming to ANC follow-up. The average monthly number of 848 pregnant women visited the hospital. The sampling interval was determined by dividing the total population who had follow-up during a month of data collection period in OPDs of the JMC ANC unit by the sample size. The selected skip interval was by taking total pregnant women of 848 and sample size 423. Therefore, the sampling fraction was 848/423 ≈ 2.

### Variables

#### Dependent variable

Sleep quality: good /poor

#### Independent variables

##### Socio-demographic factors

Age, religion, marital status, ethnicity, educational status, income status, employment, pre-pregnancy BMI, and residence.

##### Obstetrics and clinical factors

Gestational age (1st trimester, 2nd trimester, 3rd trimester), gravidity, parity, unplanned pregnancy, maternal depression, anxiety, known history of mental illness, and comorbidity of medical illness **(**like asthma, cardiovascular, diabetes, hypertension, etc. …).

##### Psychosocial factors and behavioral characteristics

Social support, stress, alcohol use, khat use, and smoking cigarettes.

#### Operational definitions

##### Sleep quality

Using a global scale PSQI good sleep quality (score = < 5) and poor sleep (score > 5) [17].

##### Depression

A total score of > 13 points using EPDS, was considered as maternal depression [18].

##### Anxiety

Anxiety was assessed using the anxiety subscale adapted from the Depression, Anxiety, and Stress Scale (DASS-21), participants who scored ≥8 were considered as having anxiety [19].

##### Past and current medical illness

Pregnant women who have known chronic medical illness and their diagnosis confirmed in any health institution either governmental or private that currently had follow-up for any chronic medical illness that was assessed by Yes or No questions.

##### Current substance use

Use of at least anyone of substance in the past three months [20].

##### Perceived stress

A cut-off value of 20 participants with a total PSS score more than or equal to 20 will be defined as stressed, meanwhile those with a score < 20 were grouped as non-stressed [21] .

##### Social support

Using the Maternity Social Support Scale (MSSS) those pregnant mothers were scored < 18 low social support, score 18–23 (medium social support), and 24–30 (strong social support) [22].

##### BMI

A person was classified as underweight (BMI < 18.5 kg/m^2^), normal body weight (BMI 18.5–24.9 kg/ m^2^), overweight (BMI 25–29.9 kg/m^2^), or obese (BMI ≥ 30 kg/m^2^) [23].

### Data collection method and tools

A structured interviewer-administered questionnaire was used, which has different subunits, questionnaires to assess socio-demographic factors, Pittsburgh sleeping quality index (PSQI), factors that affect sleep quality including obstetrical factors/clinical factors, psychosocial factors /substance use factors.

The outcome variables were assessed using the Pittsburgh Sleep Quality Index (PSQI). The PSQI has 19 items, which are categorized into seven components: subjective sleep quality, sleep latency, sleep duration, habitual sleep efficiency, sleep disturbances, use of sleeping medication, and daytime dysfunction over the last month. Each component scores ranging from 0 to 3 and then getting a global score with an interval from 0 to 21. This yields sensitivity and a specificity of 89.6 and 86.5% respectively [17]. The Cronbach alpha of PSQI in the current study was 0.78.

The Edinburgh Postnatal Depression Scale (EPDS) was used to assess the symptoms of maternal depression. It consists of 10 items and each question has four possible answers with an interval of 0–3, and a maximum score out of 30. Similar to the previous study, those who score 13 and above were considered as depressed mood [18]. EPDS is a common tool for screening depressive symptomatology; initially, for use during the postnatal periods, it is also additionally validated for use during the perinatal periods in different countries and settings The EPND is validated among the perinatal population in Ethiopia. The Cronbach alpha of EPDS in the current study was 0.91.

Adapted anxiety subscale from the Depression, Anxiety, and Stress Scale (DASS − 21) wasused to assess anxiety. Each item contributes 0 to 3 points to the sum score, resulting in a totalscore that intervals from 0 to 21, to considered anxiety, the score was 8 and above [19].

Substance use was assessed by ever use of alcohol, Khat, cigarette, and other particular substance for non-medical purposes in life. The current use of the substance was assessed for the last three months [20]. The presence of a known chronic medical such as hypertension, diabetes mellitus, or others was assessed by yes/ no response.

The perceived stress scale (PSS) was used to assess stress during pregnancy. The PSS has 10 items of self-report psychological instruments for measuring the perception of stress. Each item contains 0 to 4 points to the sum score resulting in a total score that intervals from 0 to 40, a higher score indicating greater perceived stress occurring one month before the interview [21]. Social support was assessed by the Maternity Social Support Scale (MSSS). MSSS has three categories; a score of less than 18 is low social support, 18–23 medium social support, and 24–30 high social support) [22].

### Data collection procedure

Data were collected by five trained BSc psychiatry professionals and one supervisor from the MSc student in psychiatry was also trained on how to supervise data. Each section of the questionnaire was prepared in English and then translated into the Amharic and Afan Oromo, and to ensure its understandability and consistency, then back-translated to English by an independent person. Data collectors and a supervisor have received training for two days duration on the purpose of the study, tools, how to collect data, sampling techniques, and how to handle ethical issues including confidentiality. The pretest was done among 21 (5%) of the sample size pregnant women in Agaro General Hospital before the main study was done to identify impending problems in the proposed study such as data collection tools and to check the performance of the data collectors. The supervisor and principal investigator were made regular supervision to ensure that all necessary data were appropriately collected. Each day throughout the data collection period, the completed questionnaires were assured for completeness and consistency. The collected data were edited and entered into the computer, then checked twice and processed appropriately.

### Data processing and analysis

The collected data were edited, coded, and entered into the Epi Data version 3.1, and then the data was exported to Statistical Package for Social Science (SPSS) 25.0 version for analysis. The bivariate logistic analysis was performed to select candidate variables. All variables with a *p*-value < 0.25 in the bivariate analysis were entered into the multivariable logistic regression model. Multivariable logistic regression analysis was employed to control for possible confounding effects and to determine the presence of a statistically significant association between independent variables and dependent variables. Hosmer and Lemeshow goodness was used to check the necessary assumptions. The strength of the association was presented by an adjusted odds ratio of 95% CI and *P*-value < 0.05 was considered as statistically significant. Descriptive statistics results containing frequency, percentages, and summary statistics (mean values and standard deviation) were presented to define the study population about relevant variables.

## Result

### Socio-demographic characteristics of participants

In this study, a total of 415 participants were assessed, with an overall 98.1% of response rate. The mean (SD) age of the participants was 25.22 (±4.62) years. More than half (51.8%) of respondents were Muslim religious followers. Almost three fourth of the participants (75.4%) were married and 251(60.5%) of them were Oromo in their ethnicity. One-third (32.8%) of participants have attended college and above and the majority 163(39.3%) were housewives. Most of the respondents, 320(77.1%) lived in urban areas. More than three fourth, 326(78.6%) of the participants had a normal body mass index (BMI). More than one-third of (39.8%) women reported that their average monthly income is below 1000 Ethiopian birr (Table 1).

**Table 1:** Socio-demographic characteristics of pregnant women attending antenatal care at Jimma medical center, Jimma, Southwest Ethiopia, 2020(*N* = 415)

Variables	Categories	Frequency(***n*** = 415)	Percent (%)
Age in years	18–24	200	48.2
	25–29	128	30.8
	≥30	87	21.0
Religion	Muslim	215	51.8
	Orthodox	113	27.2
	Protestant	81	19.5
	Others*	6	1.5
Marital status	Lack of cohabiting partner	102	24.6
	Married	313	75.4
Ethnicity	Oromo	251	60.5
	Amhara	69	16.6
	Yeme	35	8.4
	Keffa	29	7.0
	Gurage	20	4.8
	Others**	11	2.7
Education status	Have no formal education	32	7.7
	Primary	120	28.9
	Secondary	127	30.6
	College and above	136	32.8
Occupational status	Government employed	88	21.2
	Merchant	38	9.2
	Farming	14	3.4
	Student	67	16.1
	Housewife	163	39.3
	Private employed	45	10.8
Residence	Urban	320	77.1
	Rural	95	22.9
BMI(pre-pregnancy)	< 18.5 kg/m^2^	36	8.7
	18.5–24.99 kg/m^2^	326	78.6
	25–29.99 kg/m^2^	40	9.6
	≥30 kg/m^2^	13	3.1
Average monthly income (Eth. Birr)	≤1000	165	39.8
	1001–2000	99	23.8
	≥2001	151	36.4

Others*:-Adventist &Catholic, **:- Tigre, Wolyita & Dawro

### Obstetrics related characteristics of the participants

According to this study, nearly half of the study participants (47.0%) were in the third trimester in their gestational age. Almost two-thirds (64.8%) were multigravida and three hundred eight (74.2%) of the participants were multipara. Eighty-four participants (20.2%) had reported a previous history of abortion. More than two-thirds (68.0%) of the women had a planned pregnancy (Table 2).

**Table 2:** Description of obstetrics-related factors among pregnant women attending antenatal care at Jimma medical center, Jimma, Southwest Ethiopia, 2020(*N* = 415)

Variables	Categories	Frequency(***n*** = 415)	Percent (%)
Pregnancy by trimester	First trimester	112	27.0
	Second trimester	108	26.0
	Third trimester	195	47.0
Gravidity	Primigravida	146	35.2
	Multigravida	269	64.8
Parity	Nullipara	107	25.8
	Multipara	308	74.2
Current pregnancy status planned	No	133	32
	Yes	282	68

### Clinical related factors of the participants

According to this study finding, more than one-quarter of the participant (27.5%) and one hundred fourteen (34.0%) had comorbid depression and anxiety symptoms respectively. Twenty-one (5.1%) of respondents had a history of mental illness. From respondents, 23(5.5%) women had a comorbid medical illness, from these medical illnesses, HIV/AIDS 4(1%), asthma 5(1.2%), diabetes 6(1.4%), and hypertension 8(1.9%) were reported (Table 3).

**Table 3:** Description of clinical related factors among pregnant women attending antenatal care at Jimma medical center, Jimma, Southwest, Ethiopia, 2020(*n* = 415)

Variables	Categories	Frequency(***n*** = 415)	Percent (%)
Comorbid depression	Yes	114	27.5
	No	301	72.5
Past or current mental illness history	Yes	21	5.1
	No	394	94.9
Anxiety	Yes	141	34.0
	No	274	66.0
Chronic medical illness	Yes	23	5.5
	No	392	94.5
HIV/AIDS	Yes	4	1.0
	No	411	99.0
Asthma	Yes	5	1.2
	No	410	98.8
Diabetes	Yes	6	1.4
	No	409	98.6
Hypertension	Yes	8	1.9
	No	407	98.1

### Psychosocial and substance-related factors of the participants

From the total of the respondents, about one-third (34.7) of the respondents had stress during pregnancy. Related to social support, more than half (53.3%), 141(34.0%), and 53 (12.8%) of the pregnant women had medium social support, high social support, and poor social support respectively. Regarding the current substance use, 19(4.6%),13(3.10%), and 3(0.7%) had used alcohol, chewing khat, and smoking a cigarette within the past three months before data collection time respectively (Table 4).

**Table 4:** Psychosocial and substance factors among pregnant women attending antenatal care at Jimma medical center, Jimma, Southwest, Ethiopia, 2020(*n* = 415)

Variables	Categories	Frequency(***n*** = 415)	Percent (%)
Stress	Yes	144	34.7
	No	271	65.3
Social support	Low social support	53	12.8
	Medium social support	221	53.2
	High social support	141	34.0
Current alcohol use	Yes	19	4.6
	No	396	95.4
Current Khat use	Yes	13	3.1
	No	402	96.9
Current tobacco use	Yes	3	0.7
	No	412	99.3

### Prevalence of sleep quality among pregnant women

In the current study, the prevalence of poor sleep quality among pregnant women was 30.8% (95% CI (26.5, 35.2). Among the total respondents, 98 (23.6%) rated their overall sleep quality as bad. Below one-half (42.7%) of the respondents had faced 31–60 min sleep latency. More than two-thirds (68.9%) of the respondents had greater than 7 h of sleep duration per night. The average bedtime of the respondents was 10:05 p.m. Most of the participants, 377 (90.8%), had > 85% sleep efficacy. Almost all, 389 (93.7%) respondents had never used sleeping medication for their sleep disturbance and 82 (19.8%) of the participants reported that their sleep quality affects their day-to-day function (Table 5).

**Table 5:** Characteristics of Sleep disturbance among pregnant women attending antenatal care at Jimma medical center, Jimma, Southwest, Ethiopia, 2020(*n* = 415)

Variables	Category	Frequency (n)	Percent (%)
Subjective sleep quality	Very good (0)	180	43.4%
	Fairly good (1)	137	33.0%
	Fairly bad (2)	82	19.8%
	Very bad (3)	16	3.8%
Sleep latency	0–15 min (0)	60	14.5%
	16–30 min (1)	108	26.0%
	31–60 min (2)	177	42.7%
	> 60 min (3)	70	16.8%
Sleep duration	> 7 h	286	68.9
	6–7 h	64	15.4
	5–6 h	43	10.4
	< 5 h	22	5.3
Habitual sleep efficiency	≥85%	377	90.8
	75–84%	35	8.5
	65–74%	3	0.7
	< 65%	0	0%
Sleep disturbances	Never (0)	63	15.2%
	1 time a week (1)	306	73.7%
	1–2 times a week (2)	41	9.9%
	≥3 times a week (3)	5	1.2%
Use of sleeping medications	Never	389	93.7%
	Less than Once a week	15	3.6%
	Once or twice a week	9	2.2%
	Three or more times a week	2	0.5%
Daytime dysfunction over the last month	No problem (0)	333	80.2%
	1–2 times a week (1)	62	14.9%
	3 times a week (2)	15	3.6%
	> 3 times a week (3)	5	1.3%
Overall sleep quality	Good sleep quality	287	69.2%
	Poor sleep quality	128	30.8%

### Factors associated with sleep quality among pregnant women

Bivariate and multivariable logistic regression analysis was done to identify factors associated with sleep quality among pregnant women. On the bivariate analysis, age, marital status, educational status, residence, income, gestational age, gravidity, unplanned pregnancy, depression, anxiety, stress, social support, and alcohol use showed a *p*-value of < 0.25 and became a candidate for multivariable analysis. In multivariable binary logistic regression variables; age, gravidity, depression, and stress were found to be statistically associated with sleep quality at a p-value less than 0.05.

The odds of poor sleep among respondents with the age of older age (> = 30) was 1.94 times higher as compared to young age [18–25] [AOR = 1.94;95% CI (1.03,3.66)]. Those pregnant women with multigravidas were 1.90 times more likely to have poor sleep quality as compared with participants with primigravidas [AOR = 1.90;95%CI (1.90,3.32)]. Mothers with comorbid depression were about 4.26 times more likely to have poor sleep quality than their referent groups [AOR = 4.26;95%CI (2.54,7.14)]. Likewise, the odds of poor sleep quality among women who had stress was about 1.85 times higher as compared with their counterparts [AOR = 1.85;95%CI (1.20,3.02)] (Table 6).

**Table 6:** Bivariate and multivariate logistic regression analysis results of sleep quality among pregnant women attending ANC at JMC, Jimma, Southwest Ethiopia, 2020 (*N* = 415)

Variables	Category	Sleep quality		COR(95% C.I)	AOR(95% C.I)	***P***-values
		Poor sleep quality (n)	Good sleep quality (n)			
Age	18–24	59(29.5%)	141(70.5%)	1	1	
	25–29	30(23.4%)	98(76.6%)	0.73(0.44,1.22)	0.98(0.53,1.81)	0.95
	≥30	39(23.4%)	48(76.6%)	1.94(1.15,3.27)	1.94(1.03,3.66)	0.041*
Marital status	Single	46(45.1%)	56(54.9%)	2.31(1.45,3.68)	1.47(0.85,2.52)	0.17
	Married	82(26.2%)	231(73.8%)	1	1	
Educational status	No formal education	12(37.5%)	20(62.5%)	1.66(0.74,3.75)	0.87(0.31,2.40)	0.78
	Primary	39(32.5%)	81(67.5%)	1.34(0.78,2.29)	1.46(0.85,2.52)	0.16
	Secondary	41(32.3%)	86(67.7%)	1.32(0.78,2.25)	1.01(0.53,1.91	0.99
	College and above	36(26.5%)	100(73.5%)	1	1	
Residence	Rural	35(36.8%)	60(63.2%)	1.42(0.88,2.31)	0.97(0.54,1.73)	0.92
	Urban	93(29.1%)	227(70.9%)	1	1	
Income (Ethio birr)	≤1000	68(41.2%)	97(58.8%)	2.08(1.29,3.37)	1.71(0.98,2.97)	0.06
	1001–2000	22(22.2%)	77(77.8%)	0.85(0.46,1.55)	0.88(0.46,1.71)	0.71
	≥2001	38(25.2%)	113(74.8%)	1	1	
Current pregnancy planned	No	46(34.6%)	87(65.4%)	1.29(0.83,2.01)	0.97(0.57,1.64)	0.91
	Yes	82(29.1%)	200(70.9%)	1	1	
Gestational age	First trimester	45(40.2%)	67(59.8%)	1.75(1.07,2.86)	1.01(0.56,1.81)	0.99
	Second trimester	29(26.9%)	79(73.1%)	0.96(0.56,1.63)	0.72(0.39,1.31)	0.28
	Third trimester	54(27.7%)	141(72.3%)	1	1	0.99
Gravidity	Primigravida	31(21.2%)	115(78.8%)	1	1	
	Multigravida	97(36.1%)	172(63.9%)	2.09(1.31,3.34)	1.90(1.09,3.32)	0.025*
Depression	Yes	66(57.9%)	48(42.1%)	5.30(3.33,8.44)	4.26(2.54,7.14)	< 0.001*
	No	62 (20.6%)	239(79.4%)	1	1	
Anxiety	Yes	61(43.3%)	80(56.7%)	2.35(1.53,3.63)	1.10(0.61,1.83)	0.80
	No	67(24.5%)	207(75.5%)	1	1	
Stress	Yes	65(45.1%)	79(54.9%)	2.72(1.76,4.19)	1.85(1.20,3.02)	0.013*
	No	63(23.2%)	208(76.8%)	1	1	
Social support	Low social support	26(49.1%)	27(50.9%)	3.15(1.6,6.13)	1.32(0.60,2.87)	0.49
	Medium social support	69(31.2%)	152(68.8%)	1.48(0.92,2.41)	0.99(0.57,1.71)	0.97
	High social support	33(23.4%)	108(76.6%)	1	1	
Alcohol	Yes	13(68.4%)	6(31.6%)	5.29(1.96,14.26)	2.55(0.85,7.70)	0.10
	No	115(29.0%)	281(71.0%)	1	1	

*Statistically significant at *P*-value < 0.05, AOR, Adjusted odds Ratio, 1 = reference category, Chi square = 8, Hosmer Lemeshow goodness-of-fit 0.52, degrees of freedom = 8, Maximum VIF =1.42

## Discussion

The finding of this study showed that the prevalence of poor sleep quality among pregnant women was 30.8% [(95% CI, 26.5, 35.2)]. The study finding was higher as compared with a study done in Finland 15% [24], Peru 17% [25], {Venugopal, 2018 #34} and China 15.2% [26]. The variation might be due to the eligibility criteria and sample size. In Peru, pregnant women were between 24 and 28 weeks of pregnancy with 1298 participants while the current study was done on pregnant women including all trimesters of gestation, and the sample size was 415 [25]. Another discrepancy might be due to the difference in social support practice, sampling technique, the socio-cultural and demographic context of the women.

However, the current study was lower than the meta-analysis study conducted by Sedov et al. 45.7% [27], Turkish 86% [28], Vietnam 41.2% [29], Iran 77% [30], Taiwan 60% [31], and meta-analysis study in China 54.2% [32]. This discrepancy might be due to variation in used inclusion criteria, sampling technique, study design, and sampling size. A study conducted in Iran included third-trimester pregnancy only, and a convenience sampling technique was used while the current study used a systematic random sampling technique [30]. Another possible reason might be the difference in study design, socioeconomic, sociocultural, and demographic characteristics in the populations.

Regarding factors associated with poor sleep quality, in the current study; older maternal age was nearly two times more likely to have poor sleep during the pregnancy period as compared to younger ones. Sleep quality among pregnant women decreases as the age of the mother increases, this finding was supported by other studies done in Taskiran (Turkish) (28, 29 and 45-year-old had worse sleep quality than the age group between 17 and 28), Taiwan [33]. China [12], Vietnam [29], and Meta-analysis [27]. The possible reason might be due to usually older mothers have a tendency to have care burdens, heavier domestic responsibilities, more likely to experience physical distresses, and slower recovery from delivery, this causes a probable reason for the decline of sleep quality [34].

This finding also revealed that pregnant women with multigravidas were nearly two times more likely to have poor sleep quality than those who are primigravida. We found that poor sleep quality during pregnancy was associated with multigravidas in cross-sectional analyses. Nevertheless, our findings were inconsistent with those finding from Francisco [35] which only found an association between sleep quality and primigravida. A possible explanation might be multigravidas pregnant mothers complained that their sleep pattern is influenced by their children’s sleep habits. When children frequently wake up and/or cry at night, mothers are expected so they also wake up from their sleep to take care of theirs children.

The odds of having poor sleep quality among women who had depression were about 4.26 times higher when compared with the counterpart. This was consistent with the conducted in China [36]. This could be due to the presence of prenatal depression is one of the most possible psychological factors contributing to sleep disturbance during pregnancy [12].

The likelihood of developing poor sleep quality among pregnant women who have stress was 1.85 times higher as compared with no stress. A similar study was done in China [36]. The possible reason might be due to the direct effect of stress during pregnancy on sleep quality might be related to arginine vasopressin hormone, which is involved in the stress response and circadian regulation of the sleep-wake cycle [37].

## Conclusion

Our study found that the prevalence of poor sleep quality among pregnant women was high as compared with studies done in the general population and many other countries. Older age, multigravidas, depression, and stress were statistically significantly associated with poor sleep quality in this study. It is a good alarm to be alert to give attention to routine screening of sleep patterns in pregnant women and to give special concern for pregnant women with the above-stated factors.

## Data Availability

The datasets used and/or analyzed during the current study are available from the corresponding author on reasonable request.

## Abbreviations

AIDS: Acquired Immune Deficiency Syndrome
ANC: Antenatal Care
AOR: Odds Ratio
BMI: Body Mass Index
CI: Confidence Interval
COR: Crude odds ratio
EPDS: Edinburgh Postnatal Depression Scale
Epi-Data: Epidemiological Data
JMC: Jimma Medical Center
MDD: Major Depressive Disorder
OPD: Out Patient Department
PSQI: Pittsburgh Sleep Quality Index
SD: Standard Deviation
SPSS: Statistical Package for Social Science
USA: United States America
